# Supplementary material for: Clerodane Diterpenoids from an Edible Plant Justicia insularis: Discovery, Cytotoxicity, and Apoptosis Induction in Human Ovarian Cancer Cells
Source: Molecules. 2021 Sep 30;26(19):5933. doi: 10.3390/molecules26195933 (PMC8512812; doi:10.3390/molecules26195933)
Supplement: Supplementary file 1 [file molecules-26-05933-s001.zip › molecules-1386836-supplementary.pdf]

Supplementary Materials

Clerodane diterpenoids from an edible plant *Justicia insularis*: discovery, cytotoxicity, and apoptosis induction in human ovarian cancer cells

Idowu E. Fadayomi[1], Okiemute R. Johnson-Ajinwo[1], Elisabete Pires[2], James McCullagh[2], Tim D. W. Claridge[2], Nicholas R. Forsyth[1], and Wen-Wu Li[1*]

[1]School of Pharmacy and Bioengineering, Keele University, Stoke on Trent, ST4 7QB, UK

[2]Department of Chemistry, University of Oxford, Mansfield Road, Oxford, OX1 3TA, UK

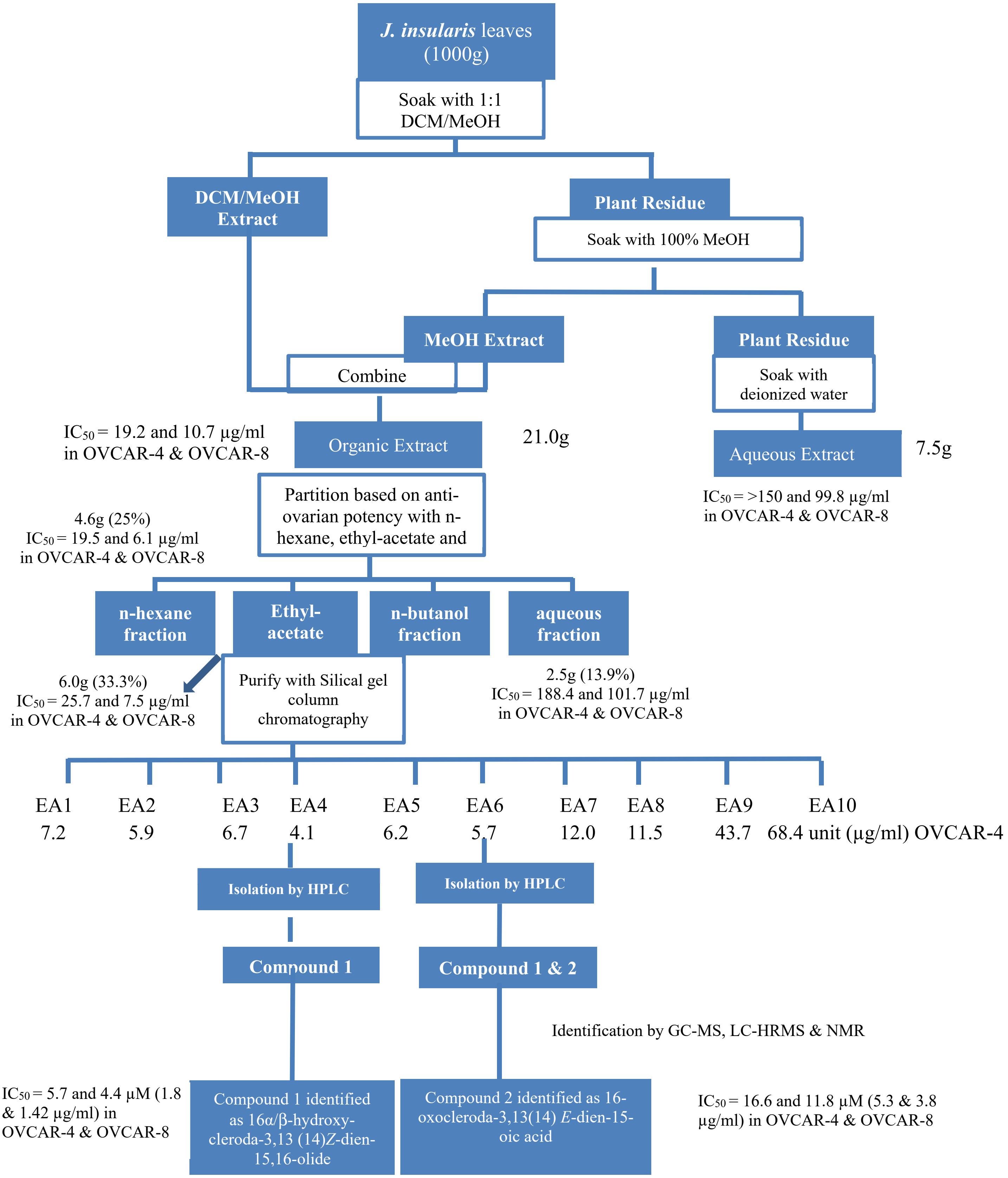

Figure S1. Scheme showing the extraction, bioassay-guided purification, and identification of cytotoxic compound **1** and 2 from *J. insularis.*

A) B)

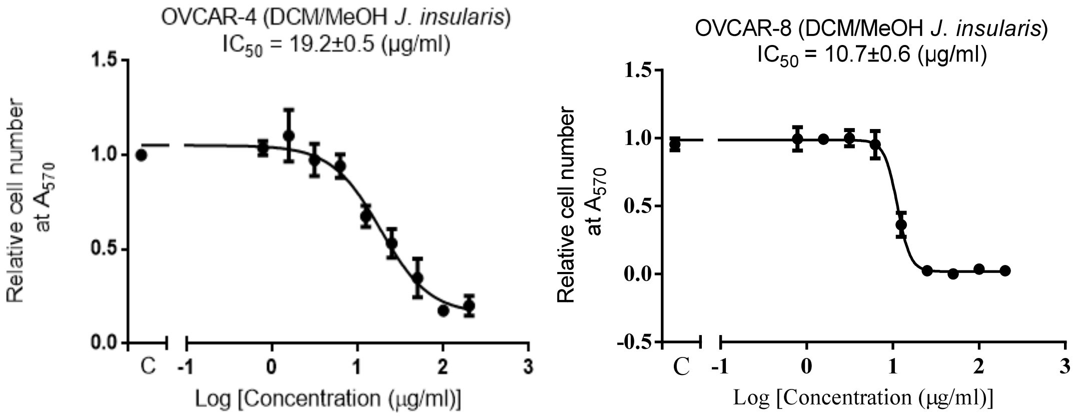

Figure S2: Mean concentration-response curve of the active extract of *J. insularis* (DCM/MeOH) in OVCAR 4 (**A**) and OVCAR 8 (**B**) ovarian cancer cell lines, showing potent cytotoxic activity of the organic extracts.

A) B)

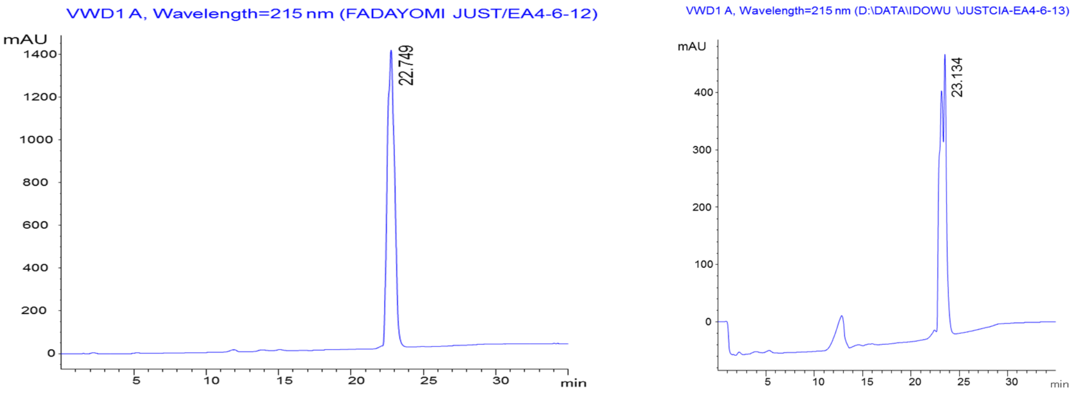

Figure S3. Analytical HPLC chromatograms of purified active compounds of *J.insularis*. (**A**) Compound **1** has a single peak at retention time 22.749 min indicating a purity of 97% and (**B**) compound **2** shows a major peak at retention time 23.134 min (purity 85%) and presence of a minor peak.

A)

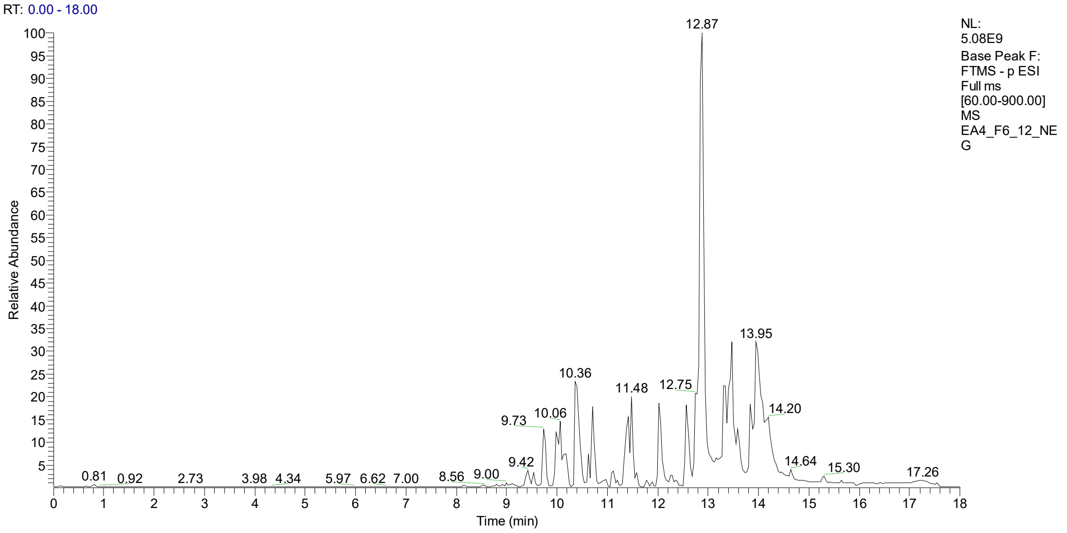

B)

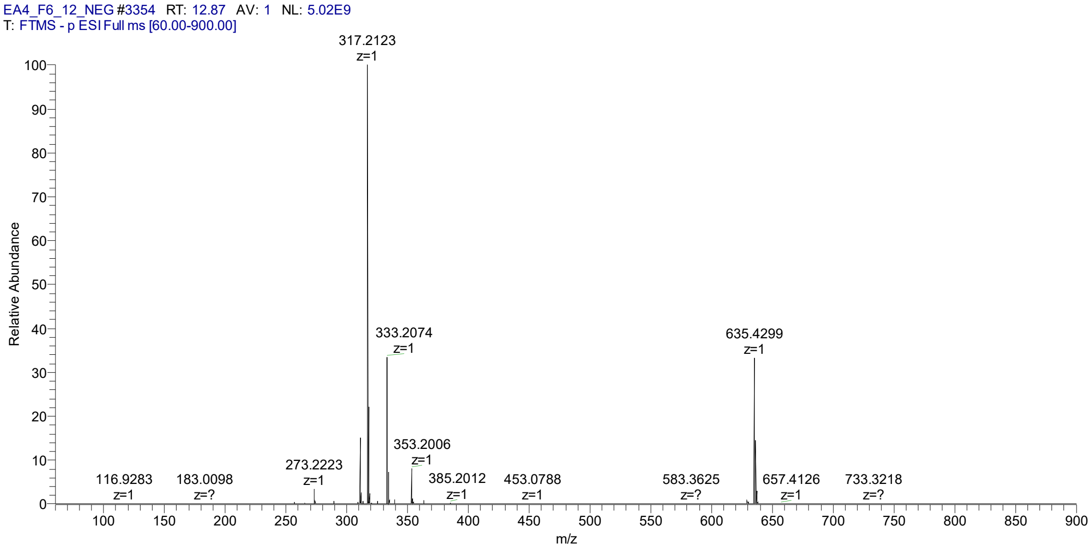

Figure S4: LC-HRMS chromatogram (**A**) and negative ESI-MS spectrum (**B**) of isolated compound **1** showing the HRMS of the major peak at a retention time of 12.87 min. The found high resolution mass of *m/z* at 317.2123 $[M-H]^-$ was consistent with its theoretical masses of 317.2117.

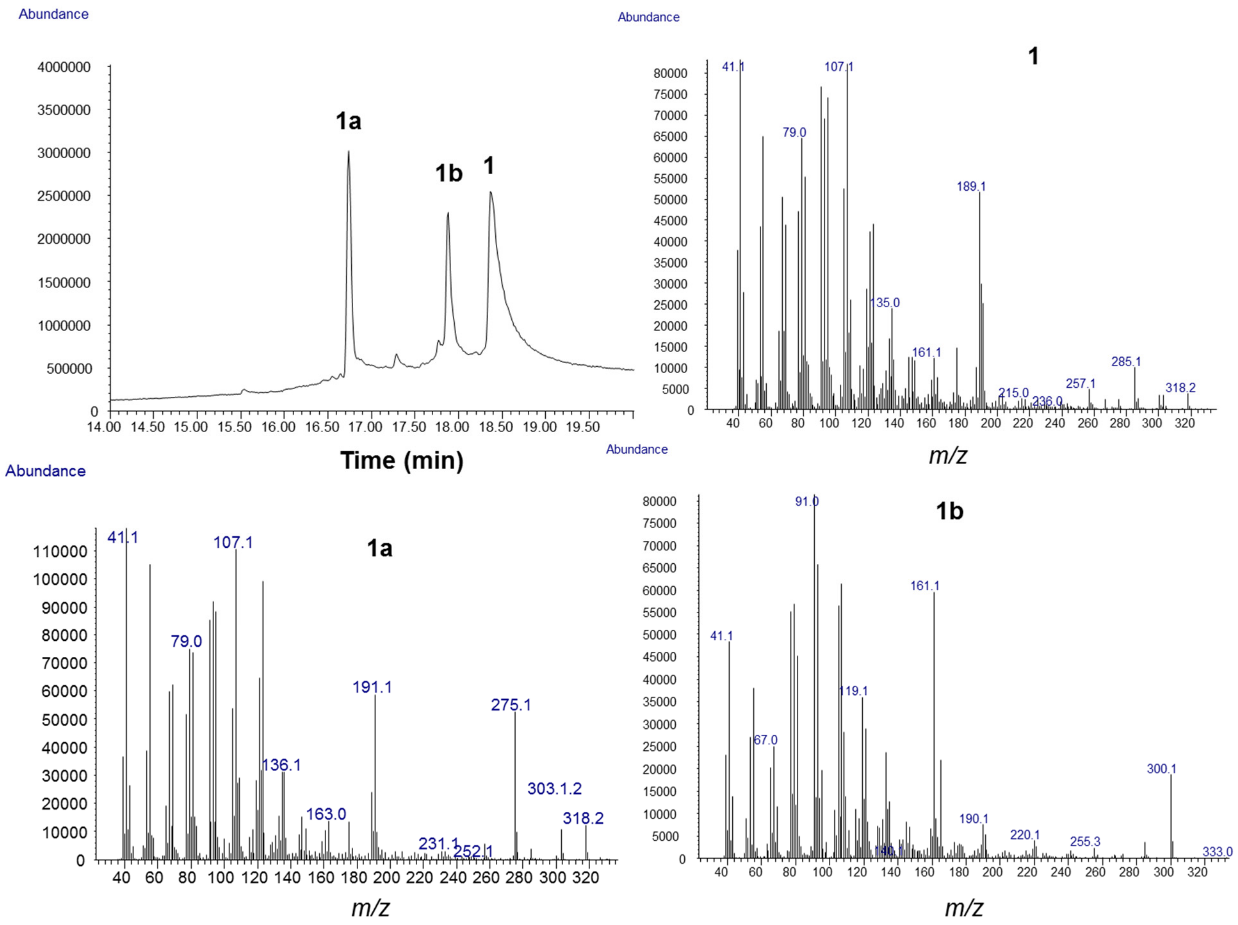

Figure S5: GC-MS chromatogram and mass spectra of the isolated compound **1** at Rt 18.45 min and two thermal degradation products of **1a** and **1b** under high temperature in the oven of GC-MS. Compound **1a** shows similar fragmentation ions as those of **1** but with an intensive ion *m/z* at 285.1 instead of 275.1 for **1**. Compound **1** and **1a** are unlikely their isomers due to the presence of this significant different fragmentation ion. Compound **1b** shows a molecule ion *m/z* at 300 due to loss of a water molecule instead of an ion at 318.2 for **1**.

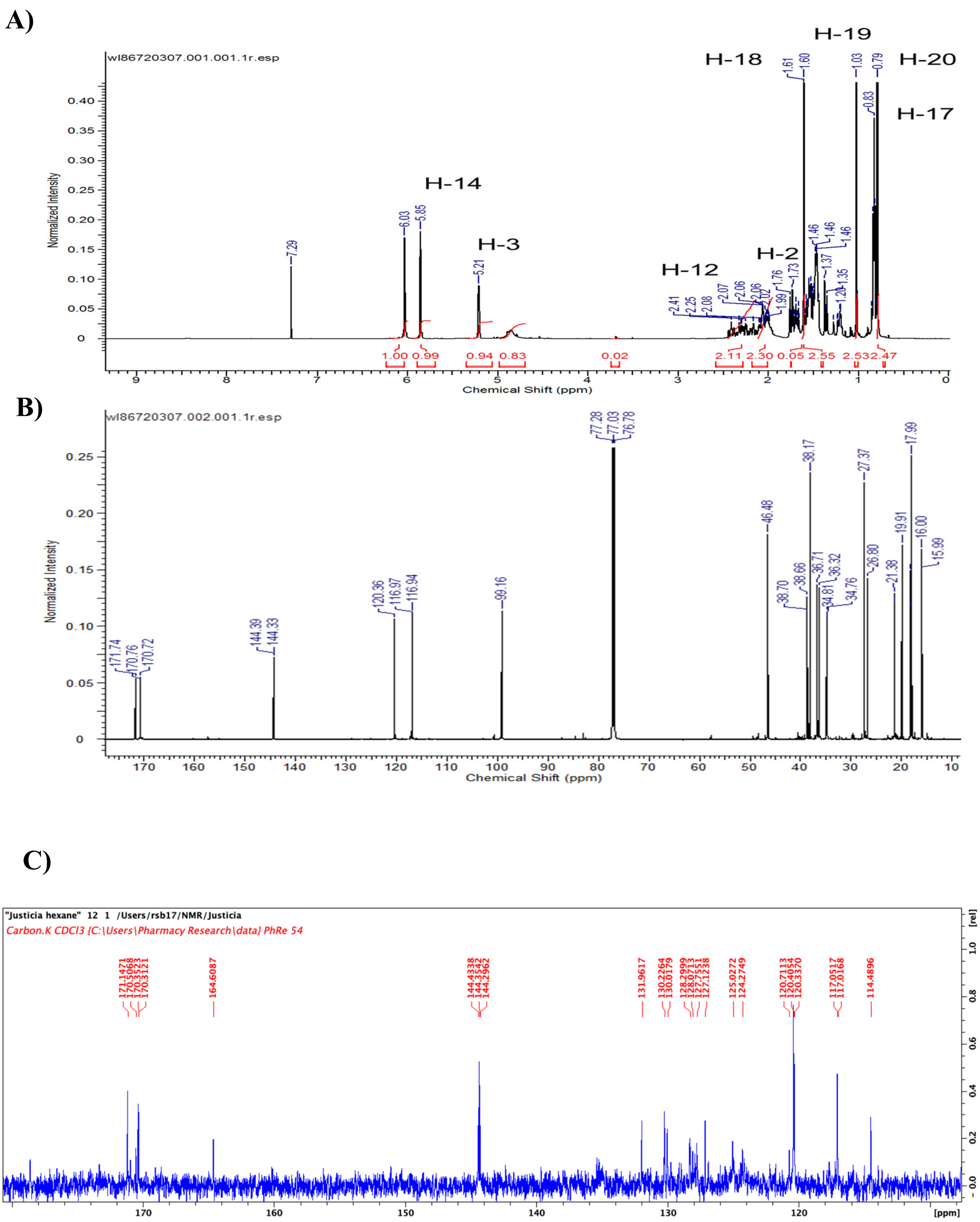

Figure S6: NMR data analysis of *Justicia insularis* of the purified compound 1 and hexane fraction in $CDCl_3$: (**A**) ^{1}H-NMR (500 MHz), (**B**) ^{13}C-NMR (125 MHz) spectrum, (**C**) ^{13}C-NMR (100 MHz) spectrum (range of 110-180 ppm) of hexane fraction containing 1 before silica gel chromatography and HPLC. The assignment of each peak is indicated in Table S3. The ratio of the 16α and 16β form of compound **1** is determined to be 1:1 by calculating the ratio of the integration value of the carbon peak at 116.94 ppm (C-14, 16α form) to that of the peak at 116.97 ppm (C-14, 16β form). The epimers of compound **1** were present in the hexane fraction before purification process which may cause isomerisation, so epimers of compound **1** are natural products in *J. inuslaris*.

A)

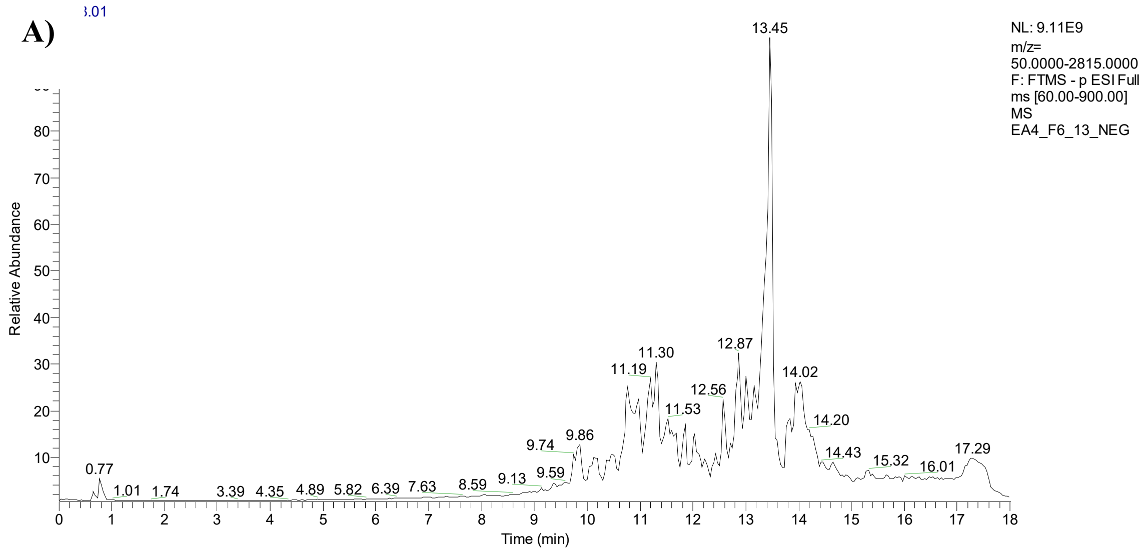

B)

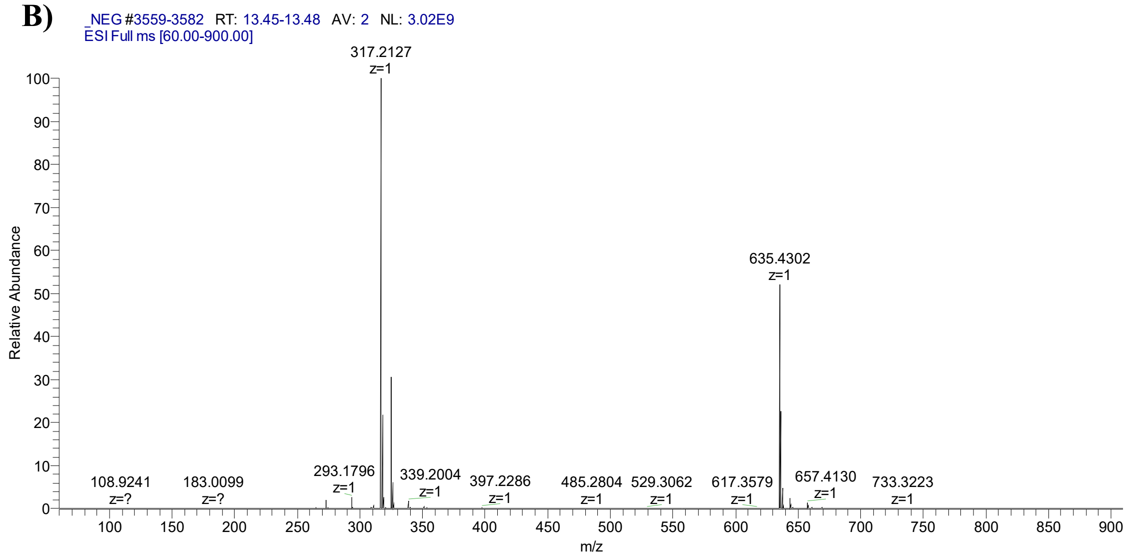

Figure S7: LC-MS chromatogram (**A**) and negative ESI-MS spectrum (**B**) of isolated compound 2 at retention time of 13.45 min. The found masses of *m/z* at 317.2127[M-H]$^-$, and 635.4302 [2M-H]$^-$ were consistent with their theoretical masses of 317.2117 and 635.4312, respectively.

A)

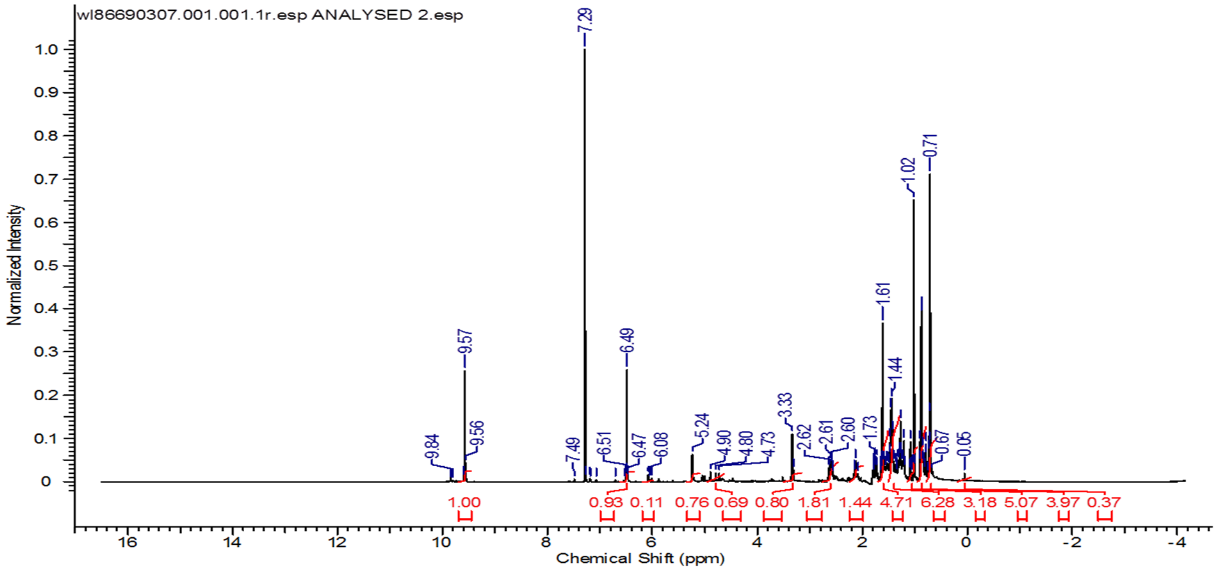

B)

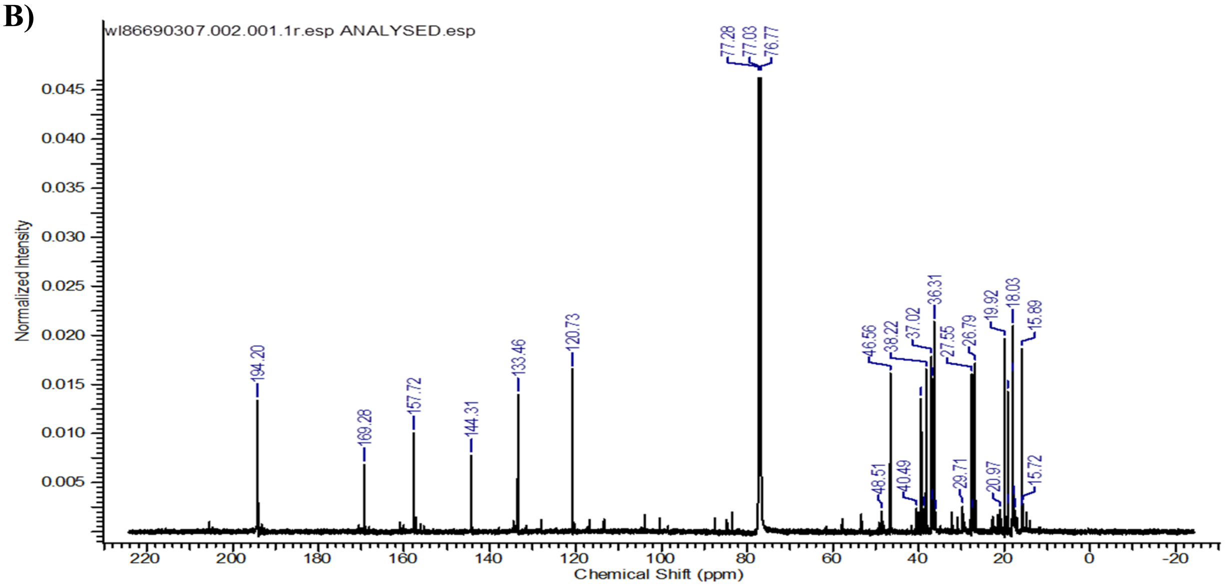

Figure S8: NMR data analysis of *Justicia insularis* purified compound 2 in $CDCl_3$: (**A**) ^{1}H-NMR and (**B**) ^{13}C-NMR. The assignment of each peak is indicated in Table S3.

Table S1: The results of the growth inhibitory activities of *J. insularis* extracts and fractions on ovarian cancer OVCAR-4 and OVCAR-8 cell lines.

Extracts, Fractions and Pure compounds	Abbrev.	IC_{50} on OVCAR-4 (µg/mL)	IC_{50} on OVCAR-8 (µg/mL)
Dichloromethane /methanol Extract	DCM/MeOH	19.2±0.5	10.7±0.6
Aqueous Extract	Aq Ext	> 150.0	99.8±0.3
n-Hexane	*n*-Hex JI	19.5±1.0	6.1±1.8
Ethyl acetate	EA	25.7±1.5	7.5±1.7
n-Butanol	*n*-But	188.4±4.6	101.7±2.2
Aqueous fraction	Aq	80.0±0.8	71.5±14.9

Table S2: The growth inhibitory activities of EA fractions and EA4 sub-fractions of *J. insularis* on OVCAR-4 ovarian cancer cell line.

Ethyl acetate fractions of *J.insularis*	IC_{50} value (µg/mL) on OVCAR-4 cell line	EA4 Sub-fractions of *J.insularis*	IC_{50} value (µg/mL) on OVCAR-4 Cell line
EA1	7.2±0.8	EA4-1	6.8±1.0
EA2	5.9±0.1	EA4-2	9.1±2.6
EA3	6.7±0.1	EA4-3	5.3±1.1
EA4	4.1±0.4	EA4-4	3.0±0.4
EA5	6.2±0.4	EA4-5	9.4±0.5
EA6	5.7±0.8	EA4-6	2.6±0.2
EA7	12.0±0.3	EA4-7	10.3±2.4
EA8	11.5±0.3	EA4-8	4.8±0.9
EA9	43.7±1.4	EA4-9	5.8±0.9
EA10	68.4±3.3	EA4-10	7.2±0.8
		EA4-11	14.2±2.3

Table S3: ^{1}H (500 MHz) and ^{13}C-NMR (125 MHz) assignments of isolated compound 1 and 2 ($CDCl_3$).

^{1}H-NMR			^{13}C-NMR		
H	**1**(16α + 16β)	**2**	C	**1**(16α + 16β)	**2**
1	1.48-1.55 (overlapped, m, 2H)	1.79 (dt, 12.8, 3.3Hz 1H); 1.46 (overlapped, 1H)	1	18.0	18.1
2	2.03 (overlapped, 2H)	2.10 (m, 2H)	2	26.79, 26.80	26.8
3	5.21 (brs, 1H)	5.24 (brs, 1H)	3	120.36, 120.43	120.7
			4	144.33, 144.39	144.3
			5	38.2	38.2
6	1.20 (td, 13.0, 4.5 Hz, 1H), 1.73 (td, 13.0, 5.0 Hz, 1H)	1.22 (m, 1H), 1.74 (td, 12.5, 3.5 Hz, 1H)	6	36.7	36.8
7	1.46 (overlapped, 2H)	1.50 (overlapped, 2H)	7	27.37	27.6
8	1.47 (m, 1H)	1.62 (overlapped, 1H)	8	36.36, 36.32	36.3
			9	38.66, 38.70	39.4
10	1.35 (dd, 12, 2.0 Hz, 1H)	1.43 (overlapped, 1H)	10	46.5	46.6
11	1.73 (dd, 14.2, 3.3 Hz, 1H); 1.68 (ddd, 14.2, 9.1, 4.9 Hz, 1H)	1.21, 1.30 (overlapped, 2H)	11	34.8	37.0
12	16α form: 2.40 (ddd, 14.2, 5.0, 1.4 Hz, H), 2.25 (ddd, 14.2, 4.3, 1.4 Hz, 1H)<br>16β form: 2.32 (ddd, 14.1, 5.0, 1.3 Hz, 1H); 2.15 (ddd, 14.1, 4.7, 1.7 Hz, 1H)	2.57 (ddd, 11.0, 5.2, 1.6 Hz, 1H); 2.30 (ddd, 11.0, 4.4, 3.7 Hz, 1H)	12	21.35, 21.38	19.2
			13	170.72, 170.76	157.7
14	5.85 (s, 1H)	6.49 (s, 1H)	14	116.94, 116.97	133.5
			15	171.7	169.3
16	6.03 (brs, 1H)	9.57 (s, 1H)	16	99.14, 99.16	194.3
17	16α form: 0.83 (d, 5.5 Hz, 3H); 16β form: 0.82 (d, 5.8 Hz, 3H)	0.87 (d, 7.3 Hz, 3H)	17	16.00	15.9
18	16α form: 1.61 (d, 3H); 16β form: 1.60 (s, 3H)	1.61 (s, 3H)	18	18.23	18.0
19	1.03 (s, 3H)	1.02 (s, 3H)	19	19.9	19.9
20	0.79 (s, 3H)	0.71 (s, 3H)	20	18.2	18.1

Table S4. Molecular descriptors and drug-likeness of compounds 1 and 2 in *J. insularis* calculated by SwissADME web tool.

Compound	Formula	Molecular weight	Num. rotatable bonds	Num. H-bond acceptors	Num. H-bond donors	Molar Refractivity	Topological Polar Surface Area (TPSA)	Log Po/w (iLOGP)	Drug-likeness (Lipinski rule)
**1**	$C_{20}H_{30}O_3$	318.45 g/mol	3	3	1	92.89	46.53 Å²	3.10	Yes
**2**	$C_{20}H_{30}O_3$	318.45 g/mol	5	3	1	94.53	54.37 Å²	2.79	Yes

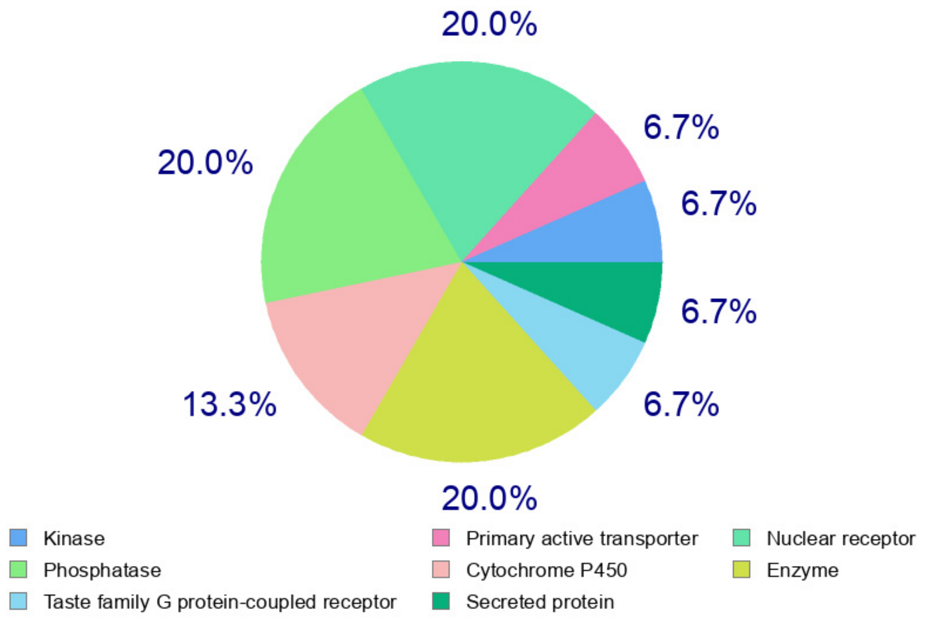

Figure S9 Distribution of predicted targets molecules of compound 1 by the SwissADME web tool (Table S5).

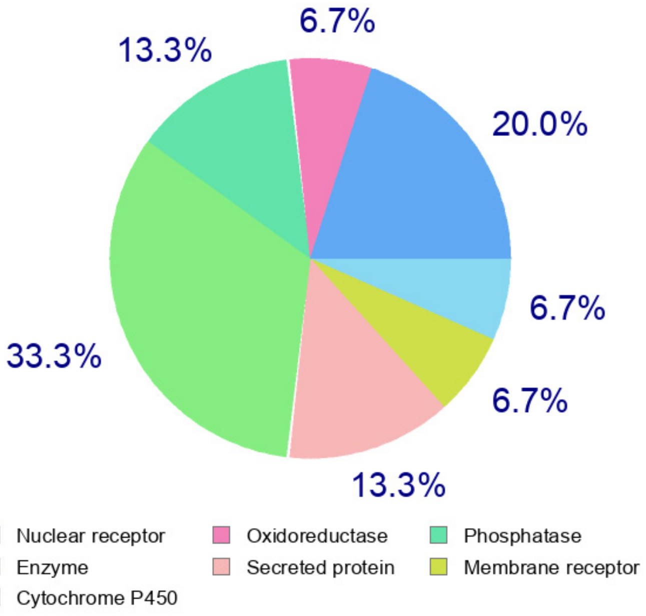

Figure S10. Distribution of predicted targets molecules of compound 2 by the SwissADME web tool (Table S6).

Table S5. Predicted protein targets of compound 1 by SwissTargetPrediction web tool (http://www.swisstargetprediction.ch).

Target	Common name	Uniprot ID	ChEMBL ID	Target Class	Probability*	Known actives (3D/2D)
Ribosomal protein S6 kinase alpha 5	RPS6KA5	O75582	CHEMBL4237	Kinase	0.13	2 / 4
Potassium-transporting ATPase alpha chain 2	ATP12A	P54707	CHEMBL2933	Primary active transporter	0.11	0 / 9
Androgen Receptor	AR	P10275	CHEMBL1871	Nuclear receptor	0.10	140 / 64
Dual specificity phosphatase Cdc25A	CDC25A	P30304	CHEMBL3775	Phosphatase	0.10	4 / 15
Progesterone receptor	PGR	P06401	CHEMBL208	Nuclear receptor	0.10	121 / 41
Cytochrome P450 19A1	CYP19A1	P11511	CHEMBL1978	Cytochrome P450	0.10	224 / 73
Dual specificity phosphatase Cdc25B	CDC25B	P30305	CHEMBL4804	Phosphatase	0.10	4 / 12
Phospholipase A2 group 1B	PLA2G1B	P04054	CHEMBL4426	Enzyme	0.10	0 / 4
Taste receptor type 2 member 31	TAS2R31	P59538	CHEMBL2034804	Taste family G protein-coupled receptor	0.10	0 / 1
Glucocorticoid receptor	NR3C1	P04150	CHEMBL2034	Nuclear receptor	0.10	80 / 12
Testis-specific androgen-binding protein	SHBG	P04278	CHEMBL3305	Secreted protein	0.10	46 / 17
Dual specificity phosphatase Cdc25C	CDC25C	P30307	CHEMBL2378	Phosphatase	0.10	0 / 4
11-beta-hydroxysteroid dehydrogenase 2	HSD11B2	P80365	CHEMBL3746	Enzyme	0.10	0 / 12
Cytochrome P450 2C19	CYP2C19	P33261	CHEMBL3622	Cytochrome P450	0.10	10 / 3
11-beta-hydroxysteroid dehydrogenase 1	HSD11B1	P28845	CHEMBL4235	Enzyme	0.10	126 / 22

Table 6. Predicted protein targets of compound 2 by SwissTargetPrediction web tool (http://www.swisstargetprediction.ch).

Target	Common name	Uniprot ID	ChEMBL ID	Target Class	Probability *	Known actives (3D/2D)
Estrogen receptor beta	ESR2	Q92731	CHEMBL242	Nuclear receptor	0.24	3 / 15
Steroid 5-alpha-reductase 2	SRD5A2	P31213	CHEMBL1856	Oxidoreductase	0.20	22 / 35
Estrogen receptor alpha	ESR1	P03372	CHEMBL206	Nuclear receptor	0.13	6 / 14
T-cell protein-tyrosine phosphatase	PTPN2	P17706	CHEMBL3807	Phosphatase	0.10	2 / 17
Protein-tyrosine phosphatase 1B	PTPN1	P18031	CHEMBL335	Phosphatase	0.10	9 / 50
11-beta-hydroxysteroid dehydrogenase 1	HSD11B1	P28845	CHEMBL4235	Enzyme	0.10	52 / 32
Nitric oxide synthase, inducible (by homology)	NOS2	P35228	CHEMBL4481	Enzyme	0.10	2 / 20
Protein farnesyltransferase	FNTA FNTB	P49354 P49356	CHEMBL2094108	Enzyme	0.10	21 / 7
Prostaglandin E synthase	PTGES	O14684	CHEMBL5658	Enzyme	0.10	25 / 13
DNA polymerase beta (by homology)	POLB	P06746	CHEMBL2392	Enzyme	0.10	0 / 9
Corticosteroid binding globulin	SERPINA6	P08185	CHEMBL2421	Secreted protein	0.10	0 / 20
Sigma opioid receptor	SIGMAR1	Q99720	CHEMBL287	Membrane receptor	0.10	0 / 3
Testis-specific androgen-binding protein	SHBG	P04278	CHEMBL3305	Secreted protein	0.10	0 / 40
Pregnane X receptor	NR1I2	O75469	CHEMBL3401	Nuclear receptor	0.10	0 / 3
Cytochrome P450 17A1	CYP17A1	P05093	CHEMBL3522	Cytochrome P450	0.10	0 / 24
Protein kinase C eta	PRKCH	P24723	CHEMBL3616	Kinase	0.10	0 / 1
Estradiol 17-beta-dehydrogenase 3	HSD17B3	P37058	CHEMBL4234	Enzyme	0.10	0 / 1

Nuclear receptor subfamily 1 group I member 3 (by homology)	NR1I3	Q14994	CHEMBL5503	Nuclear receptor	0.10	0 / 2
Dual specificity phosphatase Cdc25A	CDC25A	P30304	CHEMBL3775	Phosphatase	0.10	5 / 13
Dual specificity phosphatase Cdc25B	CDC25B	P30305	CHEMBL4804	Phosphatase	0.10	2 / 6
Leukotriene B4 receptor 1	LTB4R	Q15722	CHEMBL3911	Family A G protein-coupled receptor	0.10	17 / 6
Steroid 5-alpha-reductase 1	SRD5A1	P18405	CHEMBL1787	Oxidoreductase	0.10	7 / 8
UDP-glucuronosyltransferase 2B7	UGT2B7	P16662	CHEMBL4370	Enzyme	0.10	4 / 4
Niemann-Pick C1-like protein 1	NPC1L1	Q9UHC9	CHEMBL2027	Other membrane protein	0.10	0 / 8
Solute carrier family 22 member 12	SLC22A12	Q96S37	CHEMBL6120	Electrochemical transporter	0.10	59 / 0
Aldo-keto reductase family 1 member B10	AKR1B10	O60218	CHEMBL5983	Enzyme	0.10	13 / 6
Fatty acid-binding protein, liver (by homology)	FABP1	P07148	CHEMBL5421	Fatty acid binding protein family	0.10	1 / 3
Cytochrome P450 19A1	CYP19A1	P11511	CHEMBL1978	Cytochrome P450	0.10	1 / 297
Transient receptor potential cation channel subfamily A member 1	TRPA1	O75762	CHEMBL6007	Voltage-gated ion channel	0.10	1 / 2
Type-1 angiotensin II receptor	AGTR1	P30556	CHEMBL227	Family A G protein-coupled receptor	0.10	47 / 0
G protein-coupled receptor 44	PTGDR2	Q9Y5Y4	CHEMBL5071	Family A G protein-coupled receptor	0.10	632 / 0
Thromboxane-A synthase	TBXAS1	P24557	CHEMBL1835	Cytochrome P450	0.10	150 / 0
Progesterone receptor	PGR	P06401	CHEMBL208	Nuclear receptor	0.10	5 / 50
11-beta-hydroxysteroid dehydrogenase 2	HSD11B2	P80365	CHEMBL3746	Enzyme	0.10	5 / 21
MAP kinase ERK1	MAPK3	P27361	CHEMBL3385	Kinase	0.10	0 / 1

Arachidonate 5-lipoxygenase	ALOX5	P09917	CHEMBL215	Oxidoreductase	0.10	31 / 21
Gamma-secretase	PSEN2 PSENEN NCSTN APH1A PSEN1 APH1B	P49810 Q9NZ42 Q92542 Q96BI3 P49768 Q8WW43	CHEMBL2094135	Protease	0.10	7 / 0
Fatty acid binding protein adipocyte	FABP4	P15090	CHEMBL2083	Fatty acid binding protein family	0.10	45 / 3
Monocarboxylate transporter 1 (by homology)	SLC16A1	P53985	CHEMBL4360	Electrochemical transporter	0.10	19 / 0
Carbonic anhydrase II	CA2	P00918	CHEMBL205	Lyase	0.10	11 / 0
Carbonic anhydrase XII	CA12	O43570	CHEMBL3242	Lyase	0.10	7 / 0
Carbonic anhydrase IX	CA9	Q16790	CHEMBL3594	Lyase	0.10	7 / 0
Peroxisome proliferator-activated receptor gamma	PPARG	P37231	CHEMBL235	Nuclear receptor	0.10	144 / 16
Prostaglandin E synthase 2	PTGES2	Q9H7Z7	CHEMBL4411	Enzyme	0.10	5 / 0
TNF-alpha	TNF	P01375	CHEMBL1825	Secreted protein	0.10	0 / 6
Butyrylcholinesterase	BCHE	P06276	CHEMBL1914	Hydrolase	0.10	0 / 2
Beta-secretase 1	BACE1	P56817	CHEMBL4822	Protease	0.10	0 / 2
Arachidonate 15-lipoxygenase	ALOX15	P16050	CHEMBL2903	Enzyme	0.10	1 / 0
Peroxisome proliferator-activated receptor alpha	PPARA	Q07869	CHEMBL239	Nuclear receptor	0.10	94 / 11
Glucocorticoid receptor	NR3C1	P04150	CHEMBL2034	Nuclear receptor	0.10	9 / 34
Angiotensin-converting enzyme	ACE	P12821	CHEMBL1808	Protease	0.10	181 / 0
Neprilysin	MME	P08473	CHEMBL1944	Protease	0.10	136 / 0
Dopamine D2 receptor	DRD2	P14416	CHEMBL217	Family A G protein-coupled receptor	0.10	1 / 0
Peroxisome proliferator-activated receptor delta	PPARD	Q03181	CHEMBL3979	Nuclear receptor	0.10	28 / 9

p53-binding protein Mdm-2	MDM2	Q00987	CHEMBL5023	Other nuclear protein	0.10	72 / 0
C-C chemokine receptor type 5	CCR5	P51681	CHEMBL274	Family A G protein-coupled receptor	0.10	0 / 1
Prostanoid EP1 receptor	PTGER1	P34995	CHEMBL1811	Family A G protein-coupled receptor	0.10	264 / 7
Androgen Receptor	AR	P10275	CHEMBL1871	Nuclear receptor	0.10	3 / 92
Mineralocorticoid receptor	NR3C2	P08235	CHEMBL1994	Nuclear receptor	0.10	2 / 26
Transient receptor potential cation channel subfamily M member 8	TRPM8	Q7Z2W7	CHEMBL1075319	Voltage-gated ion channel	0.10	24 / 0
Cytochrome P450 26B1	CYP26B1	Q9NR63	CHEMBL3713687	Cytochrome P450	0.10	7 / 0
Oxoeicosanoid receptor 1	OXER1	Q8TDS5	CHEMBL1628461	Family A G protein-coupled receptor	0.10	4 / 0
Angiotensin II receptor	AGTR2	P50052	CHEMBL4607	Family A G protein-coupled receptor	0.10	4 / 0
Carboxylesterase 2	CES2	O00748	CHEMBL3180	Enzyme	0.10	0 / 14
Prolyl endopeptidase	PREP	P48147	CHEMBL3202	Protease	0.10	0 / 2
Aldose reductase	AKR1B1	P15121	CHEMBL1900	Enzyme	0.10	102 / 0
Endothelin-converting enzyme 1	ECE1	P42892	CHEMBL4791	Protease	0.10	32 / 0
Chymase	CMA1	P23946	CHEMBL4068	Protease	0.10	43 / 0
Cathepsin G	CTSG	P08311	CHEMBL4071	Protease	0.10	8 / 0
Endothelin receptor ET-A (by homology)	EDNRA	P25101	CHEMBL252	Family A G protein-coupled receptor	0.10	46 / 0
Nuclear receptor ROR-alpha	RORA	P35398	CHEMBL5868	Nuclear receptor	0.10	0 / 4

Fatty acid binding protein epidermal	FABP5	Q01469	CHE MBL3 674	Fatty acid binding protein family	0.10	8 / 1
Carbonic anhydrase I	CA1	P00915	CHE MBL2 61	Lyase	0.10	9 / 0
